# Computational Docking Reveals Co-Evolution of C4 Carbon Delivery Enzymes in Diverse Plants

**DOI:** 10.3390/ijms232012688

**Published:** 2022-10-21

**Authors:** Chao Wu, Dianjing Guo

**Affiliations:** State Key Laboratory of Agrobiotechnology, School of Life Sciences, The Chinese University of Hong Kong, Shatin, Hong Kong SAR, China

**Keywords:** carbon delivery chain, carbon fixation, PEPC, PPDK, C4 photosynthesis, protein co-evolution, co-varying sites, protein–protein interaction, computational docking

## Abstract

Proteins are modular functionalities regulating multiple cellular activities in prokaryotes and eukaryotes. As a consequence of higher plants adapting to arid and thermal conditions, C4 photosynthesis is the carbon fixation process involving multi-enzymes working in a coordinated fashion. However, how these enzymes interact with each other and whether they co-evolve in parallel to maintain interactions in different plants remain elusive to date. Here, we report our findings on the global protein co-evolution relationship and local dynamics of co-varying site shifts in key C4 photosynthetic enzymes. We found that in most of the selected key C4 photosynthetic enzymes, global pairwise co-evolution events exist to form functional couplings. Besides, protein–protein interactions between these enzymes may suggest their unknown functionalities in the carbon delivery process. For PEPC and PPCK regulation pairs, pocket formation at the interactive interface are not necessary for their function. This feature is distinct from another well-known regulation pair in C4 photosynthesis, namely, PPDK and PPDK-RP, where the pockets are necessary. Our findings facilitate the discovery of novel protein regulation types and contribute to expanding our knowledge about C4 photosynthesis.

## 1. Introduction

Proteins often interact with each other in natural states to exert their functionalities [1]. In general, interactive protein partners show similarity within their phylogenetic trees in terms of the evolutionary coupling relationship [2]. As reported in bacteria and mammals, various known proteins exhibit highly conserved interactive relationships [3,4]. In addition, due to natural selection pressure during evolution, the conservation of such interactions is retained due to non-contacting mutations [5]. From a structural point of view, the binding conformation characteristics of interactive interfaces remain untackled because only a few coupling events have been captured using co-crystallography technology. Hence, the diversity of protein–protein interactions is still largely unknown to us, especially the mutation patterns in interactive interfaces in eukaryotes.

Proteins not only interact with each other, but they also co-evolve under suitable natural selection pressure [5,6,7,8]. Among all the amino acid compositions, some residues exhibit significant and similar co-varying patterns [4,9] within single proteins and between individual proteins. The consequence of protein co-evolution is the pairwise shifts of homogeneity in specific protein families and their functional diversity [10,11]. Knowledge about protein homogeneity at various levels, such as numerical and conformational levels, is crucial for us to understand both its functional differentiation during the complex evolution process [12] and the precise anchor of its co-varying homologs.

Docking has been widely utilized to simulate the interaction between macromolecules in silico [13,14,15]. Current docking tools perform ultra-precise predictions between proteins and ligands to mimic the natural states of protein–ligand interactions. Various algorithms have been developed by computational structural biologists to provide much reliable docking information to guide protein engineering, such as antibody design and drug discovery [16,17]. Moreover, docking assists in capturing the interactive interfaces at the protein–protein level [15] and displays binding features, such as the hot-spot on the interface [18]. However, although a reliable and flexible tool, docking application was only limited to measuring the binding affinity and confirming the interface residues for further discovery and examination [19].

**Figure 1:**
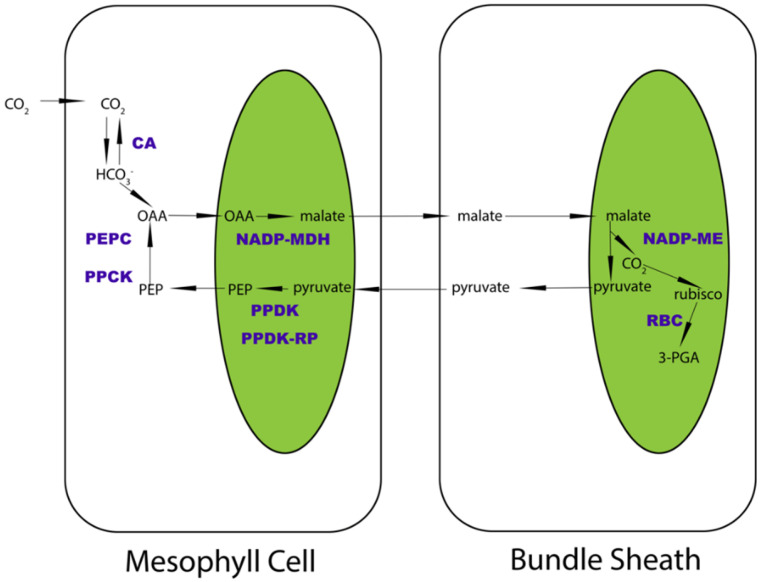
Schematic diagram of NADP-ME subtype C4 pathway. The selected eight enzymes are highlighted in purple as CA, PEPC, PPCK, NADP-MDH, PPDK, PPDK-RP, NADP-ME, and RBC. The main carbon products are shown in black. The arrows illustrate the direction of carbon transformation reactions. The green blocks show simplified chloroplasts.

In higher plants, the C4 machinery of carbon fixation and accumulation has been extensively investigated [20,21,22,23,24,25,26,27]. C4 Kranz anatomy successfully revealed the physical and biochemical differentiations of photosynthetic enzymes in two photosynthetic cells: bundle sheath and mesophyll cells (Figure 1), which are different from the ancestral C3 type. Eight key photosynthetic enzymes that deliver carbon between the two cell types have been the research focus over the years [19,28,29,30,31,32]. Research demonstrated that when atmospheric CO_2_ first diffuses into the mesophyll cells of C4 plants, it is transformed into ions by CA and initially fixed by PEPC and PPCK (the regulator of PEPC) to generate an unstable C4 acid named OAA, which is then transformed into malate by NADP-MDH and delivered into neighboring bundle sheath cells. In bundle sheath chloroplasts, malate is utilized by NADP-ME to release CO_2_ around Rubisco. This vital step vastly improves the efficiency of C4 carbon fixation, as it reduces the loss of CO_2_ in the photorespiration process. During this step, pyruvate will be synthesized, delivered back to mesophyll cells, and converted to PEP as the substrate for OAA synthesis. While in bundle sheath cells, the concentrated CO_2_ will enter the Calvin cycle to synthesize 3-PGA by RBC and finally generate organic carbon. These enzymes form a subtle carbon delivery chain to boost the efficiency of biomass accumulation, which facilitates improvement of crop yields in the grass family [33]. In contrast, few studies about the C4 carbon delivery chain have been so far reported at the protein level and from an evolutionary perspective.

Here we set out to investigate how these eight key photosynthetic enzymes evolve from C3 type to C4 type, especially whether they co-evolve at the residue level. Based on computational docking and identification of co-varying sites in each enzyme, we examine the relationship between protein–protein regulatory pairs in C4 carbon delivery chains.

## 2. Results

### 2.1. Homologous Protein Identification between C3 and C4 Species Revealed Higher Homogeneity of Photosynthetic Enzymes in C4 Plants

To catch a glimpse of the carbon delivery process in higher plants, especially in the grass family, in this study we selected eight key enzymes from C4 NADP-ME subtype grass plants. These eight enzymes were CA, PEPC, PPCK, NADP-MDH, NADP-ME, RBC, PPDK, and PPDK-RP. To carve out the phylogenetic relationship of these genes in the grass family, six representative and well-sequenced plants, including *Arabidopsis thaliana*, *Brachypodium distachyon*, *Oryza sativa*, *Setaria viridis*, *Sorghum bicolor*, and *Zea mays*, were selected for this study. 

**Figure 2:**
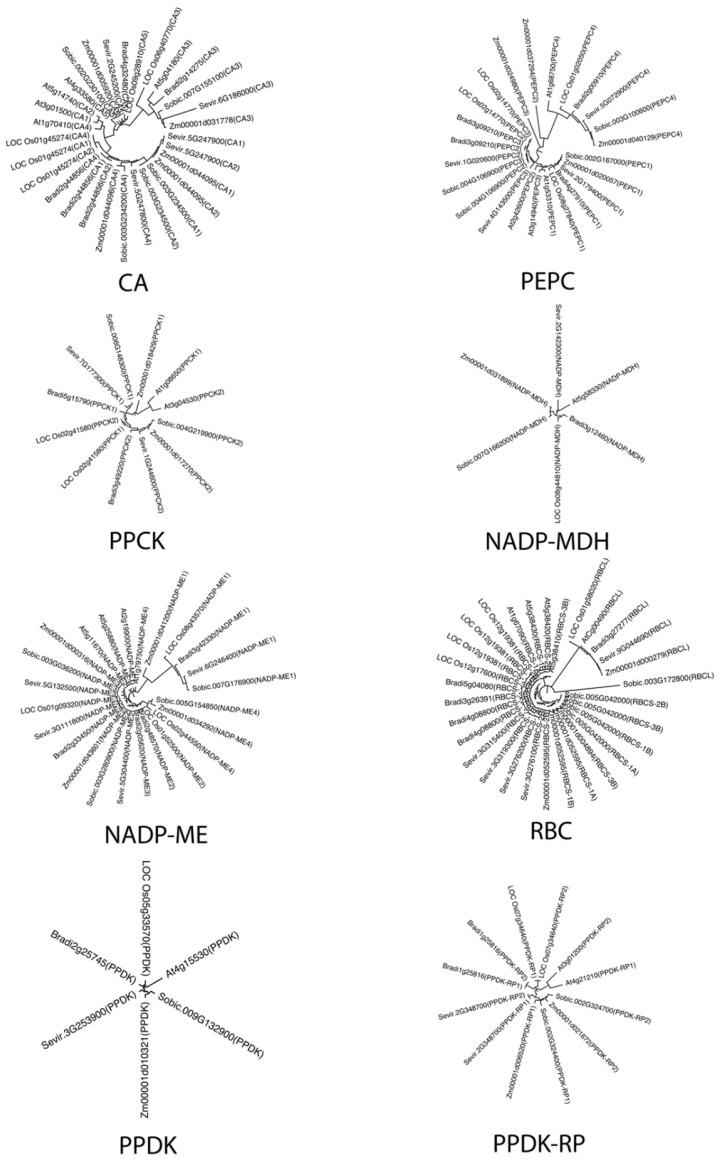
Phylogenetic trees of key enzymes in the carbon-delivery chain of C4 NADP-ME photosynthesis. Trees are arranged by the carbon transformation flow order among these enzymes. All identified paralogs and orthologs were recruited to plot the trees.

The phylogenetic trees of the homologs of each enzyme are illustrated in Figure 2. As the trees were constructed based on the amino acids sequence, we can clearly observe the relationship between each homolog at the protein level. Generally, each phylogenetic tree in the six model plants is well-illustrated in a circular format, and a similar tree structure is observed among these enzymes. The overlapped orthologs identified in the CA tree indicate that the number of CA enzymes decreased during the evolution from eudicot to monocot; it can be inferred that the essential functions of multiple CAs were replaced by fewer CA candidates. This phenomenon was not only observed in C4 plants such as Setaria viridis, Sorghum bicolor, and Zea mays, but also in the C3 plant, Oryza sativa. Since CA is the first pivotal enzyme in the whole carbon delivery chain, a CA enzyme system with high homogeneity suggests a more specific and efficient carbon capture and fixation process. This phenomenon was also observed for other enzymes, such as PEPC and RBC. Interestingly, PEPC and RBC are both used for carbon capture, with one settled in the mesophyll cells of C4 plants to fix the carbon initially and another located in the bundle sheath to undertake the major carbon fixation function. 

Higher plants evolve for improved carbon fixation efficiency, especially for those enzymes that play a pivotal role in carbon fixation. As we know, concerted evolution is the phenomenon that paralogous genes/proteins from ancestral species are differentiated and more closely related to each other in the evolved species. Generally, this phenomenon will lead to the homogenization of DNA or amino acid sequences [34]. In our study, we find the corresponding evidence at the protein level. Specifically, the sequence similarity of key photosynthetic enzymes is increased from eudicot C3 plants to monocot C4 plants (Figure 2). When comparing PEPC and PPCK for diversity of homologous proteins, we found that the plant kinases were highly conserved in terms of their functions [35]. On the other hand, based on both the co-evolution theory that a similar phylogenetic tree indicates a potential co-evolution relationship [2] and on our observation that the phylogenetic trees of PPDK and PPDK-RP are quite similar, it is reasonable to speculate that they may co-evolve.

### 2.2. N-terminus Covarying Sites Occur Distinctively in Diverse Photosynthetic Genes

**Figure 3:**
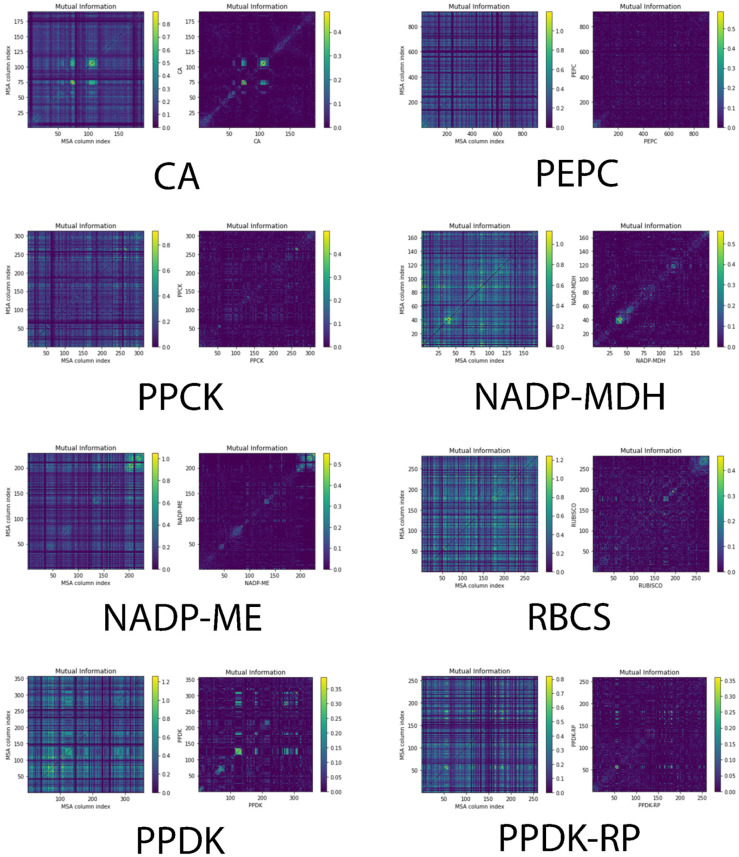
Identification of N-terminus co-varying sites in diverse C4 NADP-ME photosynthetic enzymes for carbon delivery. The sites were calculated based on mutual information theory. The left panels are the correlation of sites in the MSA format for each protein. The right panels are the heatmap diagram of these co-varying sites.

Amino acid positions constrained during evolution are presumably crucial for the protein’s structure or function, and their mutation often provides key insights into the protein’s function. These constrained amino acid positions can co-evolve inside the protein sequence or among different proteins [7,10,36]. Moreover, the N-terminus of a protein is essential for protein translation initiation and signaling in different pathways [37,38]. To investigate how the N-terminus co-evolves in each enzyme, we calculated the co-varying sites in the N-terminus of each enzyme based on multiple sequence alignment *(MSA)* and mutual information (MI) (Figure 3). For evolution studies, MSA is an efficient visualizing tool to detect the specific mutations that occur among multiple sequences. Based on information theory, MI is a measurement that calculates the mutual dependence between two input variables, which in our case was the positions of amino acids [39]. We found significant co-varying sites at the N-terminus for CA, PPDK, and PPDK-RP which are likely related to their conserved functions in carbon fixation. Co-varying sites may contribute to the self-confirmation establishment and other aspects, such as hot-spots on the interactive surface [40]. Most importantly, they may take part in protein–protein interactions. For protein-regulation pair PEPC/PPCK and PPDK/PPDK-RP, both showed a detectable level of similarity in terms of the co-varying positions of residues at the N-terminus (Figure 3). However, the co-varying positions in PPDK and PPDK-RP are clustered together in the heatmaps. We speculate that the N-terminus of PPDK and PPDK-RP is highly conserved during evolution, and those co-varying sites may be linked together to carry out important structural and functional roles.

### 2.3. Co-evolution Is Not Necessary for PEPC and PPCK to Maintain Their Regulatory Relationship

**Figure 4:**
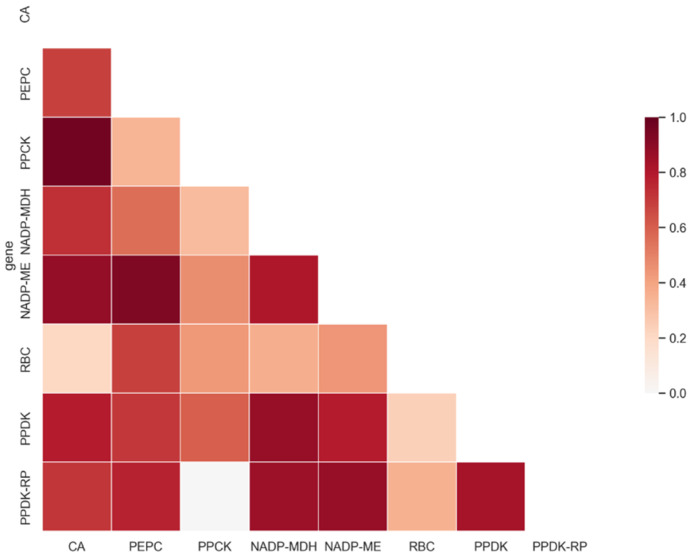
Heatmap diagram of co-evolved protein candidates calculated by Mirrortree server in carbon-delivery chain. Pairwise phylogenetic-tree-similarities measurement of selected enzymes. The red blocks show pairwise similarities. The darker the color is, the greater the tree similarity is.

Figure 4 and Supplementary Table S1 illustrate protein co-evolution based on phylogenetic tree similarity, generated by the Mirrortree server. Most photosynthetic enzymes showed high global co-evolution values over 0.5 (>30% sequence identity). Surprisingly, we found that the tree similarity score for PEPC and PPCK was only 0.339, despite that they are commonly regarded as a pair of interactive proteins. This was highly consistent with previous findings that this pair does not have similar phylogenetic trees and N-terminus co-varying sites. For PPDK and PPDK-RP, the tree similarity score was 0.831, indicating PPDK and PPDK-RP were likely correlated during the evolution process. 

As shown in Figure 3, PEPC had few co-varying sites in the N-terminus. For PPCK, although it contained more apparent co-varying sites compared to PEPC, these sites were insufficient to demonstrate a clear co-evolution relationship. To examine whether PEPC and PPCK co-evolve together we examined their phylogenetic tree similarity by using the Mirrortree server. The results of relatively low tree similarity indicated no co-evolution between these two proteins. Taken together, PEPC-PPCK regulation was different from the PPDK and PPDK-RP pair, judging by tree similarity, N-terminus co-varying sites distribution pattern, and the Mirrortree co-evolution score. Since phylogenetic tree similarity is a global measurement of protein co-evolution, Mirrortree server calculates the correlation coefficient between two different phylogenetic trees and provides global information of co-evolution between a protein pair. Apart from the known pairwise-regulation relationship in PEPC/PPCK and PPDK/PPDK-RP, we found that PEPC/NADP-ME, PPDK/NADP-MDH, NADP-ME/CA, NADP-ME/PPDK-RP, and CA/PPCK also showed rather similar phylogenetic trees (tree similarity scores of 0.936, 0.873, 0.882, 0.873, and 0.962, respectively). The overall high tree similarity scores indicate that these proteins may have a certain level of unknown interaction. We speculate that most of the photosynthetic enzymes may co-evolve together and form the C4 carbon delivery machinery and that protein–protein interaction pairs may exist widely.

### 2.4. Global Co-varying Sites Identification in C4 Enzymes

**Figure 5:**
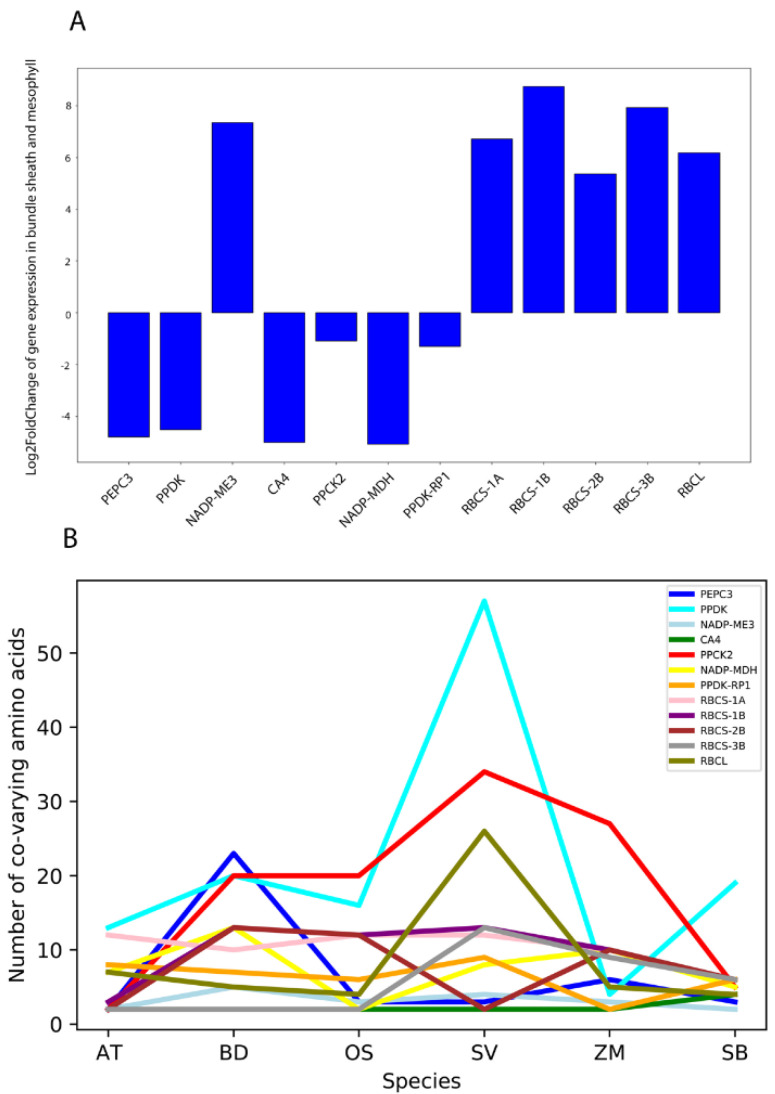
Selection of C4 candidates in carbon delivery chain and the identification of their global co-varying sites. (**A**) Differential expression patterns of selected C4 candidates that are responsible for carbon delivery in *Setaria viridis,* based on their expression preferences in bundle sheath and mesophyll cells. The blue columns represent their log2FoldChange of expression values. (**B**) Global co-varying site identification in these enzymes based on mutual information theory. The numbers of co-varying sites in each amino acid sequence are plotted for every C4 candidate. Each line illustrates the fluctuations of each C4 enzyme in six model plants.

To investigate the co-varying sites on C4 proteins of our interest, we first distinguished C4 candidates from non-C4 pathway candidates and non-photosynthetic ones based on differential gene expression between bundle sheath and mesophyll cells [41,42]. All the selected genes and their log2FoldChange values in bundle sheath vs. mesophyll cells are plotted in Figure 5A. All genes showed significant differential expression except for PPCK2 and PPDK-RP1. One reasonable explanation is that both PPCK2 and PPDK-RP1 are regulators of C4 genes that may share common regulatory functions in these two types of photosynthetic cells. In Figure 5B, global co-varying sites were calculated for each C4 photosynthetic enzyme. Surprisingly, Setaria viridis was the most distinctive species in terms of typical, sharp co-varying site distributions. Specifically, the co-varying sites of key enzymes such as PPDK, PPCK2, and RBCL were largely accumulated in Setaria viridis. From a phylogenetic perspective, Setaria viridis is considered the milestone of C4 species evolution [20,43]. The special co-varying site distribution in Setaria viridis may suggest its unique role in C4 plant evolution. 

PPDK is responsible for converting pyruvate to PEP, which enters the initial carbon fixation process in mesophyll cells. To facilitate this function, the co-varying sites in the conserved regions were retained for protein–protein interaction during evolution. PPCK2 is the regulator of PEPC. The co-varying sites in PPCK2 may serve as the conserved code for specific recognition and binding of PEPC. Rubisco is the most crucial enzyme. Located in the bundle sheath cells of C4 plants to form the C4 carbon shuttle pump (with the benefits from the C4 Kranz anatomy), it is a heteromultimer that consists of multiple RBC proteins. Among these proteins, RBCL bridges the structures generated by other RBCS proteins to form the final pose of Rubisco. The co-varying sites in RBCL may facilitate its structure maintenance and Rubisco assembly process.

### 2.5. Protein–Protein Interaction Prediction Revealed the Possible New Function of Photosynthetic Enzymes

**Figure 6:**
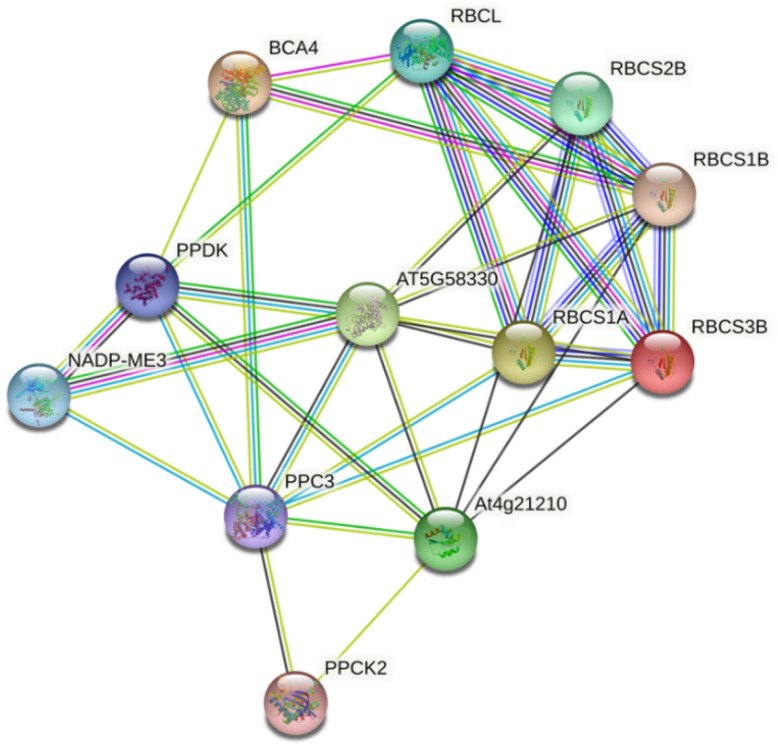
Protein–protein interaction prediction between selected C4 type of carbon delivery chain candidates in *Arabidopsis thaliana*. Each dot represents an enzyme. The lines between each dot represent the levels and confidence of the prediction. Among them, AT5G58330 is NADP-MDH and At4g21210 is PPDK-RP1.

To better understand the functions of C4 enzymes, a protein–protein interaction network was constructed for Arabidopsis thaliana using STRING (Figure 6). Regardless of the protein synthesis locations, a cross-location interaction was predicted. For example, as the first carbon capture enzyme, CA not only interacts with PPC3 (PEPC3), which is also located in mesophyll cells, but also with RBCS1B in bundle sheath cells. This makes sense, as the mesophyll and bundle sheath of C3 plant Arabidopsis thaliana do not possess the differentiation of photosynthetic functions or typical Kranz anatomy. During evolution, with the re-location of photosynthetic enzymes such as Rubisco, the protein–protein interaction may shift case-by-case in different C4 plants. 

### 2.6. Pocket Formation at the Interface Is Not Necessary for PEPC and PPCK Interaction

Pocket formation is generally regarded as the essential pose of protein–protein interaction and as a general mechanism for protein regulation during conformation packing [44,45,46]. Accordingly, amino acids show direct contact through various types of static electric forces in the pockets in order to tighten the interaction between homo- and hetero- protein candidates and to assemble them into the complex conformation [47]. Various conformation poses of interactive pockets are essential for the stability of an interactive interface, and the dynamics of pockets show the diversity of protein–protein interactions at a high resolution [48]. 

**Figure 7:**
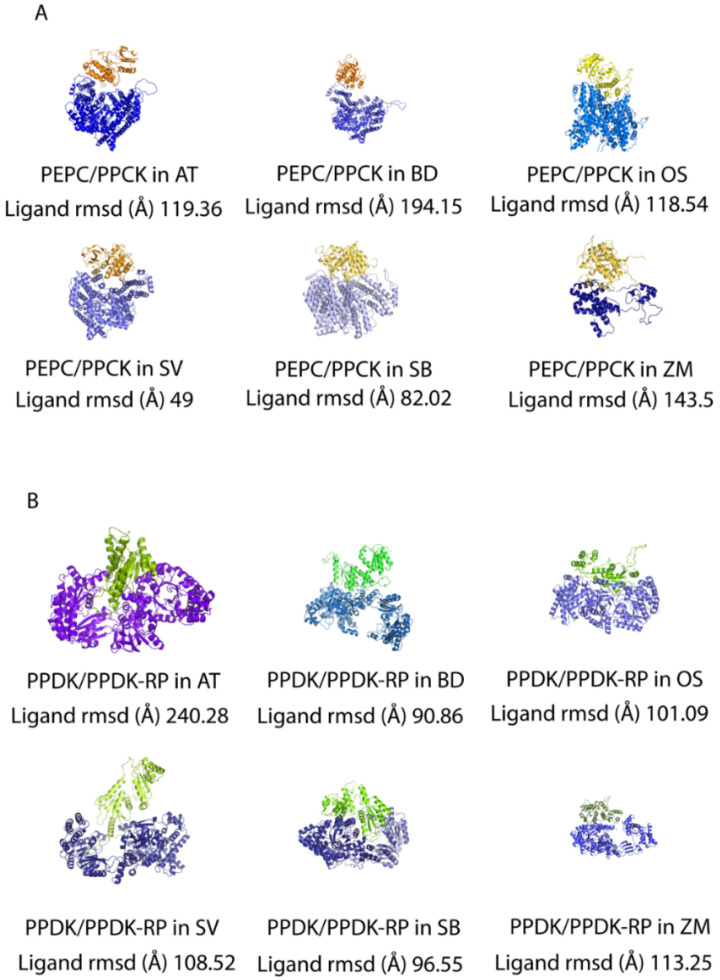
Protein–protein docking between interactive enzyme pairs. (**A**) Protein–protein docking between PEPC3 and PPCK2 in *Arabidopsis thaliana*, *Brachypodium distachyon*, *Oryza sativa*, *Setaria viridis*, *Sorghum bicolor*, and *Zea mays*. The affinity between the interaction of two proteins is illustrated by ligand RMSD values. (**B**) Protein–protein docking between PPDK and PPDK-RP1 in *Arabidopsis thaliana*, *Brachypodium distachyon*, *Oryza sativa*, *Setaria viridis*, *Sorghum bicolor*, and *Zea mays*. The affinity between the interaction of two proteins is demonstrated by ligand RMSD values as well.

Structural fluctuations of homologous proteins often happen during evolution [49]. In this study, we observed that the conformations of two regulation pairs are different in selected model plants, especially for the pocket regions, which differ drastically in different plants. We also found that pocket is not necessary for PPCK to regulate PEPC in diverse model plants. However, for PPDK and PPDK-RP pairs, the conformations of binding pockets varied case by case. Specifically, the interactive residues were tightly embedded in the pocket for Arabidopsis thaliana, while they were loosely embedded in other C3 species (Figure 7). When we examined the distances between the two proteins using ligand RMSD values as measurements based on protein docking data, we found that this protein pair showed closer interaction in C4 plants compared to C3 plants. Moreover, despite the ligand RMSD value, it was found that the PEPC/PPCK regulation pair without pocket formation was conserved from C3 to C4 plants. For the PPDK/PPDK-RP pair, pocket conformations were formed in both C3 and C4 plants. Interestingly, we also observed that some intrinsic disordered residues were embedded in the pockets (Figure 7B). Although these residues may potentially be important for site recognition and pocket formation, due to the lack of conformation data of their interaction partners, the three-dimensional structures of intrinsic disordered residues are not determined. It is worth investigating in the future whether PPDK-RP may recruit more partners to form a more complex and stable regulation pose to maintain its regulatory functions in the carbon-delivery chain machinery.

## 3. Discussion

Mutual information (MI) is a classic measurement to quantify the dependence between two random variables [50]. In protein co-evolution studies, it has been proved as the most effective computational principle widely used to design tools for mining co-evolution scenarios from the large corpus of protein data [5,7]. The original design of MI considers phylogenetic relationships, whereas a corrected version was developed with the absence of phylogenetic information [39]. In this study, we chose the original MI to calculate the inter-protein and within-protein residue co-evolution because the phylogenetic relationship is necessary for us to understand protein co-evolution, by providing a global view. In our study, we first examined the phylogenetic relationship, as it is a strong indicator of inter-protein co-evolution, according to a previous study by Gregory B. Gloor et al. [51]. Instead of sampling diverse kinds of proteins, we focused only on a group of highly conserved enzymes enriched in the C4 carbon delivery chain. On the other hand, our investigations of co-varying site distribution in the N-terminus of each selected C4 enzyme are essential for us to understand its role in C4 machinery assembly. As reported, the N-terminus is crucial for protein targeting into proper organelles [52,53]. In our study, we detected several co-varying sites enriched as clusters in the N-terminus of these photosynthetic proteins. These clustered co-varying sites were likely associated with protein targeting of the chloroplast. However, due to the lack of appropriate tools, our observations were insufficient to provide functional annotation of the identified co-varying sites.

Although our study has limitations in demonstrating the functional co-evolution in all domains of a single protein, it still provided comprehensive information for protein–protein co-evolution of C4 enzymes. For example, frequent inter-protein co-evolution is indicated (Figure 4) in this study. Such findings may provide a new strategy for engineering a group of interactive C4 proteins for improved photosynthesis in C3 plants [22]. Understanding protein co-evolution will also facilitate the selection of appropriate gene cascades to minimize the difficulty of stable gene transfer. For example, according to Kaisa Kajala et al., PEPC and PPDK do not show cell-specific expression in maize [54]. However, we observed that these two genes showed preferential mesophyll expression in *Setaria viridis* (Figure 5A). Besides, a previous study revealed the co-varying sites inside Rubisco but did not consider its association with other photosynthetic enzymes [55], especially for the regulation pairs of photosynthetic proteins. 

In our study, we selected six major crops with typical C4 traits and focused on key photosynthetic enzymes, such as Rubisco, and the regulation pairs, such as PEPC/PPCK and PPDK/PPDK-RP. We found that all RBCS proteins contained fewer co-varying sites compared to RBCL. In another study focusing on Rubisco, conducted by Mingcong Wang et al. [55], researchers calculated the co-varying sites by chi-square and identified more co-varying sites. Interestingly, we found that the intra-protein co-varying site distribution for C4 enzymes in *Setaria viridis* was drastically diverged from other C4 species, indicating that this plant species may play a unique role in C4 evolution. Meanwhile, identification of protein-binding partners, such as small molecules like H2O, may reveal the regulation machinery and facilitate our understanding of the protein function domains and the role of co-varying sites [56]. 

Although we have identified the co-varying sites in the selected C4 enzymes, whether these sites contribute to the functions of these enzymes remains elusive. For now, we retrieved the MSA from the Pfam database. Other protein databases containing various sources of information may also be taken into consideration, e.g., Uniprot for protein subcellular localization and Interpro for protein domain. Integrating this information may facilitate our understanding of the specific functions of co-varying sites. In addition, computational pipelines and tools to predict how a single mutation of amino acid residue may change the conformation of key functional regions will provide useful information for experimental validation and protein engineering in C3 crops.

## 4. Materials and Methods

### 4.1. Phylogeny Study of Eight Key Photosynthetic Enzymes

Eight key photosynthetic enzymes were selected for this study [32]. Among them, six are crucial for carbon delivery in C4 photosynthesis, except for PPCK and PPDK-RP, which are the regulators of PEPC and PPDK, respectively. Homologous proteins were selected based on our custom synteny study and from the Phytozome tool of Joint Genome Institute (https://jgi.doe.gov/) (accessed on 18 October 2022). All amino acid sequences were retrieved from the latest genome accessions, which were *Arabidopsis thaliana* TAIR10 [57], *Brachypodium distachyon* V3.1 [58], *Oryza sativa* V7.0 [59], *Setaria viridis* V2.1 [43], *Sorghum bicolor* V3.1.1 [60], and *Zea mays* RefGen V4 [61]. Selected amino acid sequences of each homologous protein were first aligned by MEGA X [62] using the MUSCLE algorithm to generate the MSA file for phylogeny plot, and the phylogeny trees were calculated by the bootstrap method set at 500 replications as default for each enzyme. The original trees were transformed into a circular format using TreeViewer (https://treeviewer.org/) (accessed on 18 October 2022).

### 4.2. Co-Evolved Positions Identification

Protein co-evolution sites were identified by ProDy (http://prody.csb.pitt.edu/) (accessed on 18 October 2022) and Evol [63] for each candidate enzyme. Using MSA calculation based on the Pfam database, we generated lists of reliable protein co-evolved residues and identified their position within the amino acids sequence. The Evol software focused on the N-terminal of protein candidates and provided comprehensive plots to visualize the protein co-evolution sites at the N-terminal end.

### 4.3. Global Tree Similarity Comparison between Eight Key Photosynthetic Enzymes

To compare the co-evolution relationship between each enzyme candidate, we used the Mirrortree server [2,64] to evaluate the phylogenetic tree similarity and to provide a similarity score to illustrate the co-evolution relationship. Mirrortree server is a classic (the first interactive assessment of protein co-evolution) method widely adopted for protein–protein co-evolution analysis by giving a correlation coefficient value [8,65,66]. It provides global analysis based on multiple homologous protein sequences of one protein query. The tree similarity values were plotted by R version 4.0.4 (https://www.r-project.org/) (accessed on 18 October 2022).

### 4.4. Selection Criteria of Key C4 Enzyme Candidates

For each enzyme, multiple homologs were collected for this study. Two criteria were applied in the selection: sequence similarity from Joint Genome Institute (https://jgi.doe.gov/) (accessed on 18 October 2022) and cell-type specific gene expression level based on their functions. High-similarity sequences were selected through comparison with C3 ancestor *Arabidopsis thaliana,* based on the best hit. To select the candidates which may participate in the C4 pathway of carbon delivery, we selected the differentially expressed candidates between bundle sheath and mesophyll cells in C4 plant *Setaria viridis,* since theoretically they are differentially expressed in these two types of photosynthetic cells.

### 4.5. Protein–Protein Interaction Prediction

To predict the possible protein–protein interaction between each selected C4 candidate, we used STRING (https://string-db.org/) (accessed on 18 October 2022). [67] database to calculate the interaction, based on the sequence input data retrieved in *Arabidopsis thaliana*. Because the protein functions are highly conserved between different homologs, we can infer the interaction in C4 plants based on the results. The results generated by STRING were considered as the interaction network among these proteins.

### 4.6. Co-Varying Amino Acids Identification

To identify the exact amino acids which were co-varying during evolution, we used Coeviz2 (https://research.cchmc.org/CoevLab/) (accessed on 18 October 2022). [68] to calculate the precise residues, with parameters set as ‘Mutual information’, ‘2-alphabet’, and ‘Pfam’. Multiple sequence alignment was generated based on the Pfam database. The numbers of co-varying sites were calculated and plotted with python NumPy (https://numpy.org/) (accessed on 18 October 2022). and Matplotlib (https://matplotlib.org/) (accessed on 18 October 2022). libraries.

### 4.7. Protein–Protein Docking

We used the HDOCK server (http://hdock.phys.hust.edu.cn/) (accessed on 18 October 2022). [15] to generate the protein–protein docking conformation to observe if the docking pocket was generated and how it was precisely illustrated. HDOCK accepted sequences as input and utilized template-based rigid body docking. The visualization of docking results was performed by PyMOL (https://pymol.org/2/) (accessed on 18 October 2022).

## 5. Conclusions

In this work, we examined the global protein co-evolution relationship and local dynamics of co-varying site shifts in eight key C4 enzymes. We found that: (i) Compared to C3 plants, homologs of key photosynthetic enzymes showed higher homogeneity in C4 plants. Based on concerted evolution theory, an increased homogeneity often reveals functional replacements of C3 ancestral proteins in C4 plants. (ii) For the selected enzymes, it was common for their N-terminus sites to co-evolve. Such phenomena may be associated with the functional aspects of the N-terminus. (iii) For PEPC and PPCK pairs, neither a co-evolution relationship nor binding pockets were necessary to maintain their regulatory relationship. (iv) Among the six model plants examined, Setaria viridis contained the most co-varying sites in each candidate enzyme, likely indicating its distinctive role in C4 photosynthesis evolution. Our work describes the complexity of protein co-evolution and regulation in C4 plants and provides a potential foundation for further investigation on PEPC and PPCK regulation mechanisms.

## Data Availability

Not applicable.

## Supplementary Materials

The following supporting information can be downloaded at: https://www.mdpi.com/article/10.3390/ijms232012688/s1.

## Abbreviations

CA	carbonic anhydrase
PEPC	phosphoenolpyruvate carboxylase
PPCK	phosphoenolpyruvate carboxylase kinase
NADP-MDH	NAD(P)+-dependent malate dehydrogenase
NADP-ME	NADP-dependent malic enzyme
RBC	ribulose bisphosphate carboxylase
PPDK	pyruvate, phosphate dikinase
PPDK-RP	pyruvate, phosphate dikinase regulatory protein
OAA	oxaloacetic acid
PEP	phosphoenolpyruvate
3-PGA	3-phosphoglyceric acid
DEG	differentially expressed gene
CO2	carbon dioxide
RMSD	root-mean-square deviation
AT	*ARABIDOPSIS THALIANA*
BD	*Brachypodium distachyon*
OS	*Oryza sativa*
SV	*Setaria viridis*
SB	Sorghum bicolor
ZM	*Zea mays*
